# Identification of transcription factors MYC and C/EBPβ mediated regulatory networks in heart failure based on gene expression omnibus datasets

**DOI:** 10.1186/s12872-020-01527-9

**Published:** 2020-05-27

**Authors:** Haiwei Wang, Xinrui Wang, Liangpu Xu, Hua Cao

**Affiliations:** Fujian Maternity and Child Health Hospital, Affiliated Hospital of Fujian Medical University, FuZhou, 350001 FuJian China

**Keywords:** Heart failure, C/EBPβ, MYC, Networks, Metabolism signaling pathway, Insulin signaling pathway

## Abstract

**Background:**

Heart failure is one of leading cause of death worldwide. However, the transcriptional profiling of heart failure is unclear. Moreover, the signaling pathways and transcription factors involving the heart failure development also are largely unknown. Using published Gene Expression Omnibus (GEO) datasets, in the present study, we aim to comprehensively analyze the differentially expressed genes in failing heart tissues, and identified the critical signaling pathways and transcription factors involving heart failure development.

**Methods:**

The transcriptional profiling of heart failure was identified from previously published gene expression datasets deposited in GSE5406, GSE16499 and GSE68316. The enriched signaling pathways and transcription factors were analyzed using Database for Annotation, Visualization and Integrated Discovery (DAVID) website and gene set enrichment analysis (GSEA) assay. The transcriptional networks were created by Cytoscape.

**Results:**

Compared with the normal heart tissues, 90 genes were particularly differentially expressed in failing heart tissues, and those genes were associated with multiple metabolism signaling pathways and insulin signaling pathway. Metabolism and insulin signaling pathway were both inactivated in failing heart tissues. Transcription factors MYC and C/EBPβ were both negatively associated with the expression profiling of failing heart tissues in GSEA assay. Moreover, compared with normal heart tissues, MYC and C/EBPβ were down regulated in failing heart tissues. Furthermore, MYC and C/EBPβ mediated downstream target genes were also decreased in failing heart tissues. MYC and C/EBPβ were positively correlated with each other. At last, we constructed MYC and C/EBPβ mediated regulatory networks in failing heart tissues, and identified the MYC and C/EBPβ target genes which had been reported involving the heart failure developmental progress.

**Conclusions:**

Our results suggested that metabolism pathways and insulin signaling pathway, transcription factors MYC and C/EBPβ played critical roles in heart failure developmental progress.

## Background

Heart failure is a rapidly growing public health issue and one of leading cause of death [1]. Serial vicious cycles of cardiomyocyte depletion, cardiac dilatation and mechanical dysfunction are culminating in heart failure [2]. Once patients have developed to the end stage of heart failure, intervention is limited to heart transplantation [3]. In order to understand the molecular mechanisms regulating heart failure, several studies have used microarrays for genome wide analysis of heart failure [4–6]. Transcriptional genomics results revealed that FOX families of transcription factors were associated with human heart failure [4]. The different mRNA splicing [5] and long non-coding RNA (lncRNA) [6] in diseased hearts was also comprehensively studied using gene microarrays. However, due to the complexity of genetic and epigenetic abnormality of heart failure, the previously reported gene expression signature in failing heart tissues is varied considerably from study to study, making it difficult to reconcile their findings or reach any definite conclusions [7]. Moreover, the mis-regulated molecular signaling pathways and key transcription factors in heart failure are largely unknown.

Transcription factors control the transcriptional activity of multiple target genes by binding to a specific region of the DNA sequence [8]. It has been reported that transcription factor C/EBPβ plays central roles in physiologic hypertrophy and heart failure [9]. C/EBPβ could repress cardiomyocyte growth and proliferation. Reducing C/EBPβ expression exaggerates the cardiac failure upon pressure overload [9]. TP53 is another major transcription factor in cardiac transcriptional network [10]. TP53 deficient hearts are resistant to the failure development upon acute pressure overload [11]. Interestingly, both C/EBPβ and TP53 are involving tumor developmental progress by regulating metabolism [12, 13] and TGFβ signaling pathway [14, 15].

MYC is an oncogene. High level of MYC expression is required for tumor initiation, progression and maintenance [16]. MYC regulates multiple critical cellular functions, for example, metabolism [17] and RNA splicing [18]. Inhibition of MYC by BET bromodomain inhibitor is a promising anti-cancer strategy [19]. Interestingly, transcriptional pause release in heart failure was mediated by BET bromodomain [20], and BET bromodomain inhibitors could suppresses the development of heart failure by the regulation of the innate inflammatory network [21]. All those results suggest the potentially significant roles of MYC in heart failure. However, the expression of MYC and MYC mediated downstream target genes are not studied in failing heart patients.

In the present study, using published GEO datasets, we tried to identify the signaling pathways and transcription factors associated with heart failure. We also tried to determine the MYC and C/EBPβ mediated downstream target genes and construct the complex transcriptional networks regulated by MYC and C/EBPβ in heart failure developmental progress.

## Methods

### Data collection

Gene expression series matrix of failing heart tissues and normal heart tissues was downloaded from GEO website (https://www.ncbi.nlm.nih.gov/geo/) with GEO number GSE5406, GSE16499 and GSE68316.

### GEO data processing

All the expression datasets were processed separately using R software (version 3.5.0; https://www.r-project.org/). The matrix file of each dataset was annotated with corresponding platform. A probe was removed if it was not corresponded gene symbol, and the expression values were averaged if multiple probes corresponded to the same gene symbol using R software “plyr” package (version 1.8.5; https://cran.r-project.org/web/packages/plyr/index.html). Plyr package includes multiple tools for splitting, applying and combining data. The different gene expression between failing heart tissues and normal heart tissues was determined using Student’s t test.

### Venn diagrams

Venn diagrams were generated using VENNY 2.1 software (http://bioinfogp.cnb.csic.es/tools/venny/index.html). VENNY 2.1 is an interactive tool for comparing lists.

### Kyoto encyclopedia of gens and genomes (KEGG) signaling pathway enrichment analysis

KEGG signaling pathways and transcription factors analysis was performed using The Database for Annotation, Visualization and Integrated Discovery (DAVID) website (version 6.8; https://david.ncifcrf.gov) [22]. DAVID is a functional annotation tool for list of genes. Enrichment *P*-value and Benjamini false discovery rate (FDR) were generated. Enriched signaling pathways and transcription factors with *P*-value < 0.05 was considered to be statistical significant.

### Gene set enrichment analysis (GSEA)

GSEA was performed using GSEA 2.0 software [23]. Signaling pathways gene sets and transcription factor targets gene sets were downloaded from the GSEA Web site (http://www.broad.mit.edu/gsea/index.html). Genes ranked by signal-to-noise ratio, and statistical significance was determined by 1000 gene set permutations. The results of significance should meet the criteria of P-value< 0.05.

### Heatmap presentation

Heatmaps were created by R software “pheatmap” package (version 1.0.12; https://cran.r-project.org/web/packages/pheatmap/). “pheatmap” is a R package offering more dimensions and appearance of heatmaps. The clustering scale was determined by “average” method. The clustering distance was determined by the ‘correlation’ method. Other parameters were provided in the usage of the “pheatmap”.

### Spearman correlation

Spearman correlation was used to study the correlation between C/EBPβ and MYC expression by the “lm” method of R software. “lm” method was used for linear regression analysis in R. *P*-value< 0.05 suggested the significant correlation between C/EBPβ and MYC expression.

### C/EBPβ and MYC associated transcriptional network

The networks of C/EBPβ and MYC downstream target genes were created by Cytoscape GeneMANIA App. Cytoscape is an open source software platform for constructing complex networks and could be download from Cytoscape website (https://cytoscape.org/). Node degrees represent the power of the connection between the selected genes.

### Statistical analysis

The box plots were generated from GraphPad software Prism8. GraphPad Prism8 was provided by GraphPad Company (https://www.graphpad.com/). Statistical analysis was performed using the two-tailed paired Student’s t test using R software. R software (version 3.5.0) was provided by The R Project (https://www.r-project.org/). *P* value less than 0.05 was chosen to be statistically significant difference.

## Results

### The transcriptomic features of heart failure

**Fig. 1:**
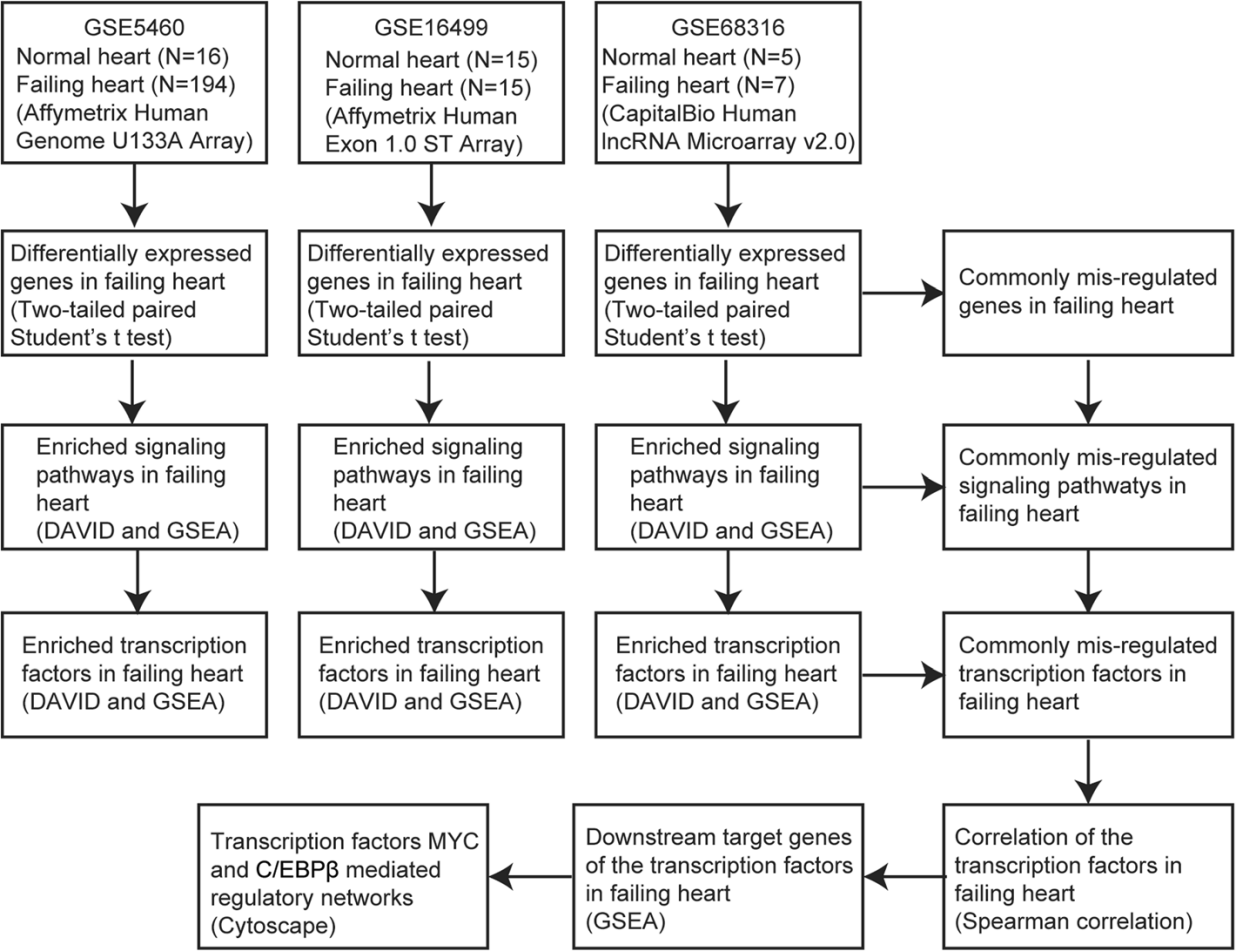
Search strategies used for accessing the gene datasets was described in the flowchart

To identify the differentially expressed genes and the critical signaling pathways and transcription factors during the development of heart failure, we analyzed the expression data of failing heart and normal heart tissues from previously published GEO datasets GSE5460 [4], GSE16499 [5] and GSE68316 [6]. Totally, 252 samples were collected, including 36 normal heart tissues and 216 failing heart tissues. The search strategies used for accessing the gene datasets were described in the flowchart (Fig. 1).

**Fig. 2:**
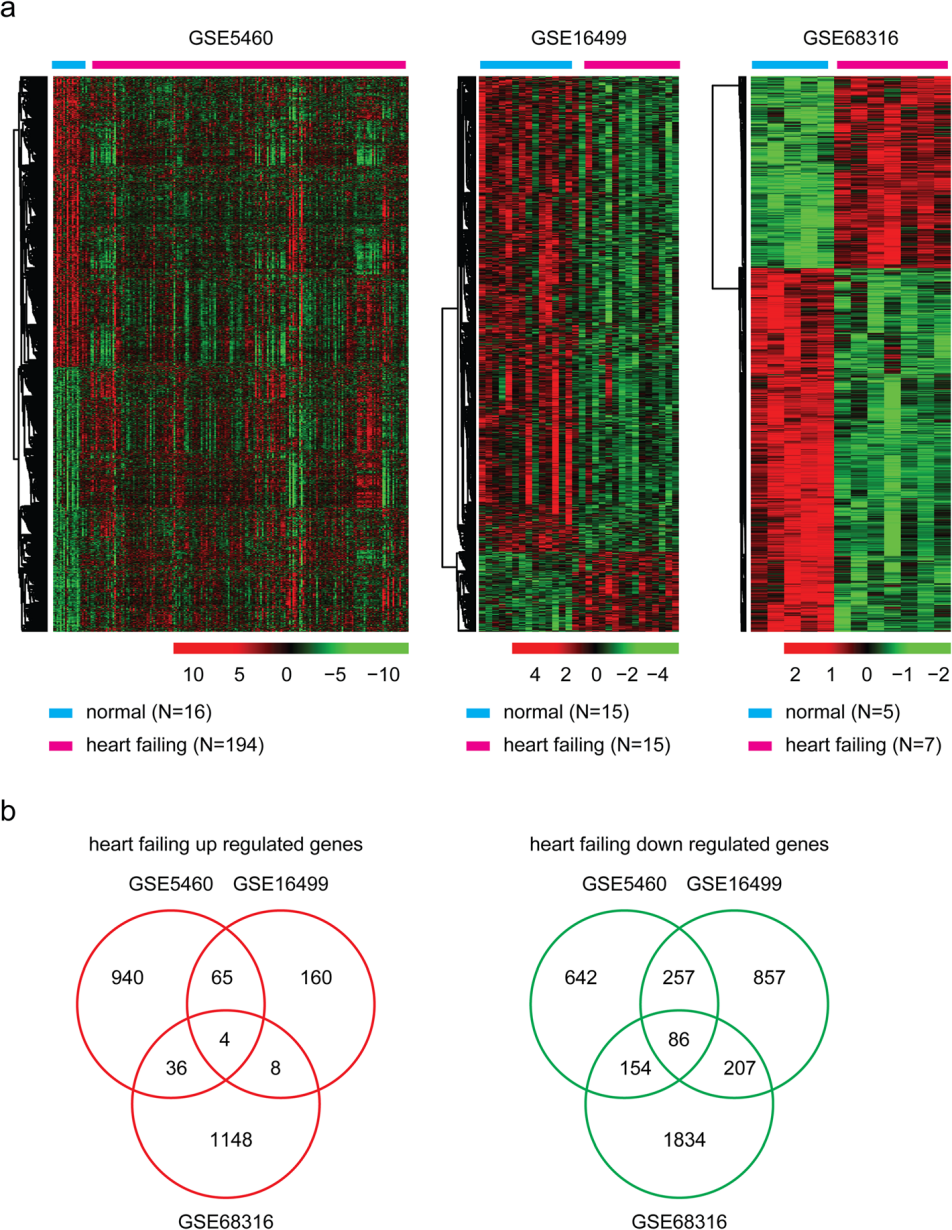
Identification of the transcriptomic features of heart failure. **a** Un-supervised clustering heatmaps represented the differentially expressed genes (*P* < 0.01) in failing heart tissues compared to normal heart tissues in GSE5406, GSE16499 and GSE68316 datasets. Each column represented one sample. Genes up-regulated (red), down-regulated (blue) and moderately regulated (black) were delineated. Number of sample in each dataset was also showed. **b** Venn diagrams depicted the overlapped differentially expressed genes in GSE5406, GSE16499 and GSE68316 datasets. Red cycle represented the up regulated genes in failing heart tissues and green cycle represented the down regulated genes in failing heart tissues

First, we analyzed the globe expression profiling of failing heart tissues in each dataset. Compared with the normal heart tissues, the differentially expressed genes in failing heart tissues (*P* < 0.01) were selected for further studies. This resulted in the identification of 2184 differentially expressed genes in GSE5406, 1644 differentially expressed genes in GSE16499 and 3477 differentially expressed genes in GSE68316 dataset (Fig. 2a). Among all the differentially expressed genes, only 4 genes were commonly up regulated and 86 genes were commonly down regulated in GSE5406, GSE16499 and GSE68316 datasets (Fig. 2b). In GSE16499 and GSE68316 datasets, the number of down regulated genes was for more than the up regulated genes (Fig. 2a). In GSE16499 dataset, 1407 genes were suppressed in failing heart tissues. While, only 237 genes were activated in failing heart tissues. Those results suggested that the depletion of cardiomyocytes and loss of mechanical functions in cardiac remodeling were induced by the suppression of heart specific genes.

### Metabolism and insulin signaling pathway are suppressed in failing heart patients

**Fig. 3:**
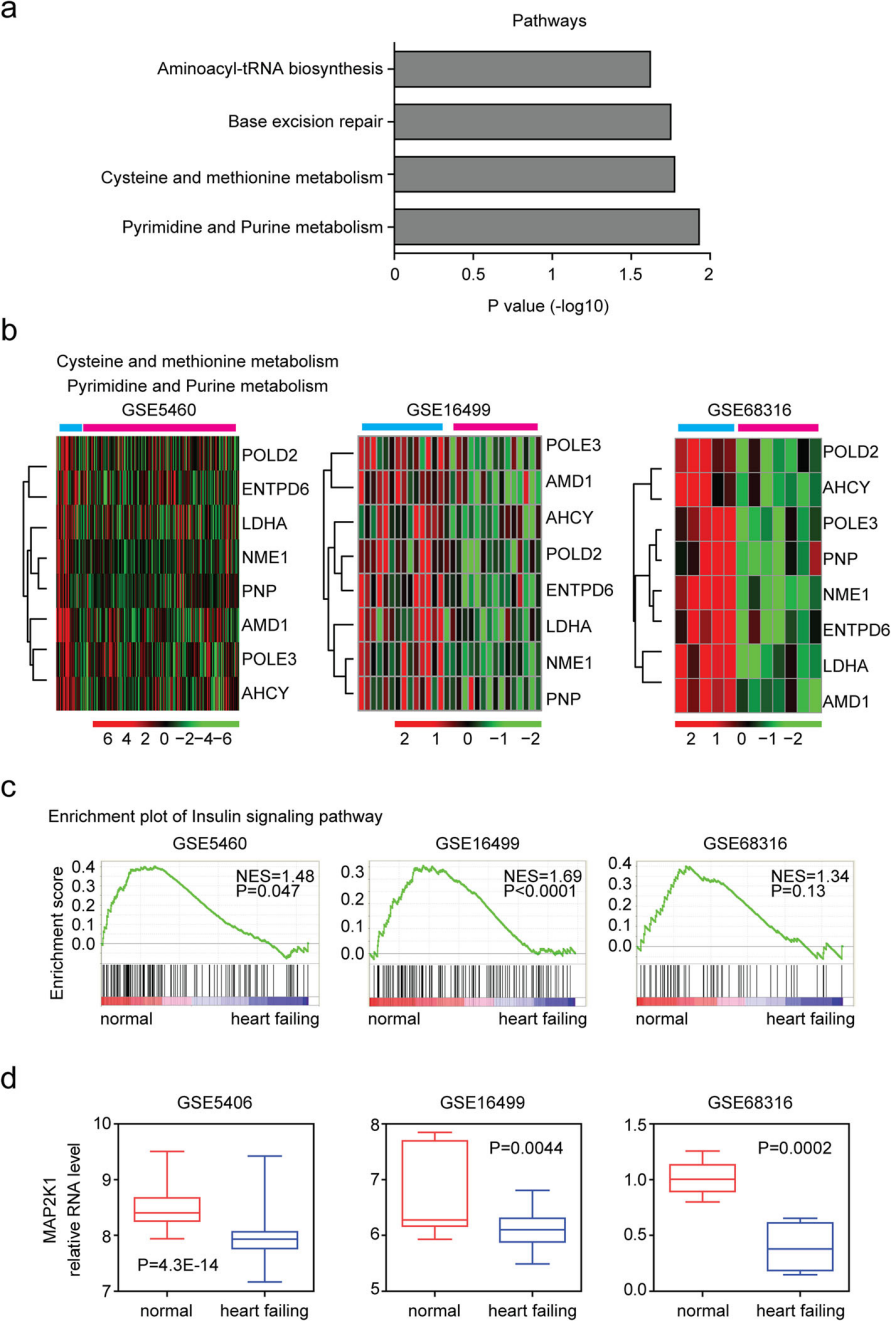
Metabolism and insulin signaling pathway are suppressed in failing heart patients. **a** Functional pathways enrichment analysis of the common differentially expressed 90 genes in failing heart tissues. **b** Differentially expressed genes from cysteine, methionine, pyrimidine and purine metabolism signaling pathway were represented using heatmaps. **c** Enrichment plots showed the enriched insulin signaling pathway in GSE5406, GSE16499 and GSE68316 datasets. Enrichment of NES and *P* values were shown. **d** Box plots showed the expression levels of MAP2K1 in GSE5406, GSE16499 and GSE68316 datasets. P values showed the difference of MAP2K1 expression levels between failing heart tissues and normal heart tissues determined by Student’s t test

To reveal the functional relevance of the common differentially expressed genes in failing heart tissues, we performed functional signaling pathway enrichment analysis through DAVID [22] and GSEA [23] assay. Pyrimidine, purine metabolism signaling pathway and cysteine, methionine metabolism signaling pathway were highly enriched through DAVID analysis (Fig. 3a). Heatmap presentations showed that NME1, POLE3, POLD2, ENTPD6, PNP genes from pyrimidine, purine metabolism signaling pathway and LDHA, AHCY, AMD1 genes from cysteine, methionine metabolism signaling pathway were all down regulated in failing heart tissues in GSE5406, GSE16499 and GSE68316 datasets (Fig. 3b), suggesting the suppression of those pathways in the development of heart failure.

Through GSEA analysis, we found that the insulin signaling pathway was negatively correlated with the failing heart expression profiling (Fig. 3c), suggesting the inactivation of insulin signaling pathway in the development of heart failure. Fox example, MAP2K1 is a critical downstream gene of insulin signaling pathway [24]. We showed that MAP2K1 was down regulated in failing heart tissues in GSE5406, GSE16499 and GSE68316 datasets (Fig. 3d).

The association between heart failure, inactivation of metabolism pathways and insulin resistance was well established [24]. The cardiac metabolism, growth and survival in the heart were dependent on insulin signaling pathway [25]. Loss of insulin signaling pathway induced cardiac energy deficiency and accelerated the heart failure progress [26]. All those observations confirmed the enriched singling pathways derived from the GEO datasets.

### Transcription factors MYC and C/EBP are negatively associated with in failing heart expression profiling

**Fig. 4:**
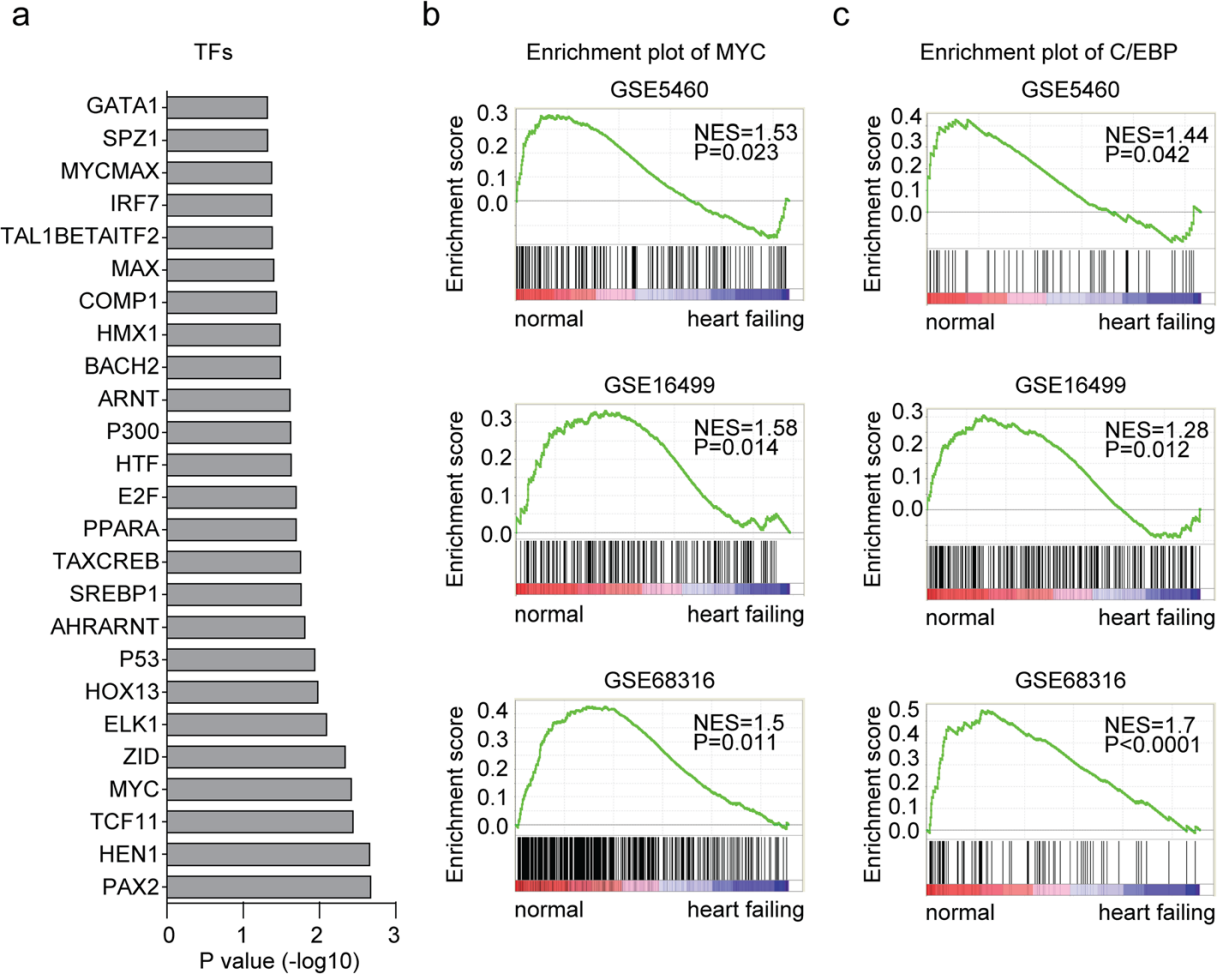
Transcription factors MYC and C/EBP are negatively associated with in failing heart expression profiling. **a** Functional transcription factors enrichment analysis of the common differentially expressed 90 genes in failing heart tissues. **b** Enrichment plots of transcription factor MYC in GSE5406, GSE16499 and GSE68316 datasets. Enrichment of NES and P values were shown. **c** Enrichment plots of transcription factor C/EBP in GSE5406, GSE16499 and GSE68316 datasets

Except signaling pathways, the transcription factors enriched in failing heart tissues were also identified through DAVID analysis. We found that transcription factor MYC was highly associated with the differentially expressed genes in GSE5406, GSE16499 and GSE68316 datasets (Fig. 4a). Interestingly, TP53 and E2F genes were both highly enriched (Fig. 4a). TP53 and E2F family genes were reported to mediate the cardiac growth and development [27]. However, the functions of MYC in the development of heart failure are unclear.

Similar results were obtained using GSEA assay. We found that transcription factor MYC was negatively associated with the failing heart expression profiling in all three GEO datasets (Fig. 4b). Additionally, we showed that transcription factor C/EBP was also negatively correlated with the failing heart expression profiling (Fig. 4c).

C/EBP is a CCAAT/enhancer-binding protein transcription factor which regulates cell growth and differentiation. Previous results showed that C/EBPβ protected against pathological cardiac remodeling [9]. C/EBPβ was also a master regulator of metabolism pathways and insulin resistance [12]. All those reports implied the potential roles of C/EBPβ in the development of heart failure.

### Transcription factors MYC and C/EBPβ are down regulated in failing heart tissues

**Fig. 5:**
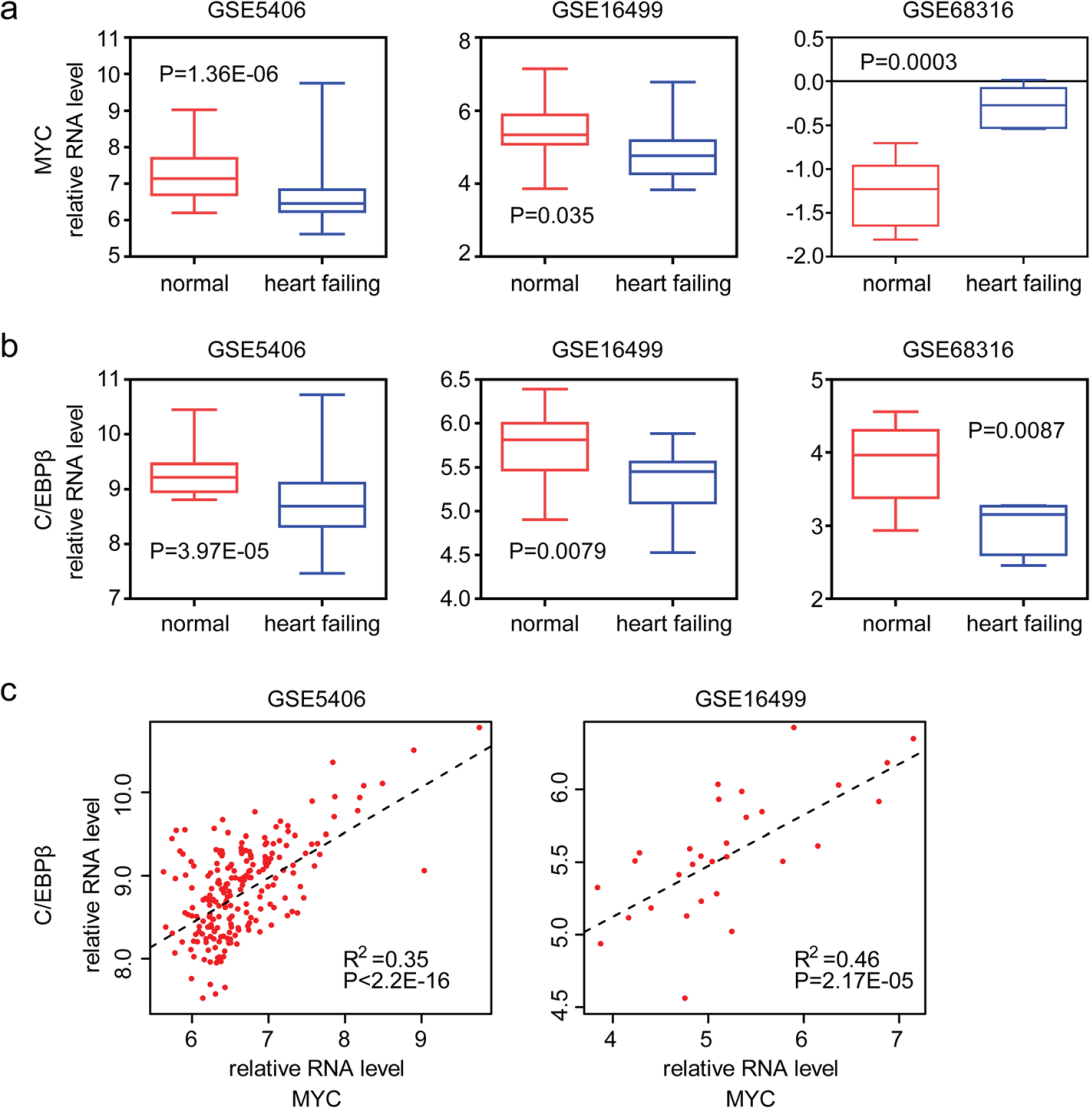
Transcription factors MYC and C/EBPβ are down regulated in failing heart tissues. **a** Box plots showed the transcription factor MYC expression levesl. P values showed the difference of genes expression levels between failing heart and normal heart tissues determined by Student’s t test. **b** Box plots showed the transcription factor C/EBPβ expression levels. **c** Pearson correlation between MYC and C/EBPβ expression levels in GSE5406 and GSE16499 datasets. Adjusted R-square and *P* value were shown

Next, we detected the expression of MYC and C/EBPβ in failing heart and normal heart tissues. Previous report showed that MYC was increased in pathological hypertrophy [28]. Inhibition of MYC was a potential therapeutic approach in the treatment of hypertrophic cardiomyopathy [29]. On the contrary, we found the down regulation of MYC expression in failing heart tissues in GSE5406 and GSE16499 datasets (Fig. 5a). Similarly, we found that C/EBPβ was down regulated in failing heart tissues, compared with normal heart tissues in all GSE5406, GSE16499 and GSE68316 datasets (Fig. 5b).

Since MYC and C/EBPβ were both down regulated in failing heart tissues, we tested the correlation between MYC and C/EBPβ expression in GSE5406 and GSE16499 datasets. We found that C/EBPβ expression was positively correlated with MYC expression. Heart tissues with high C/EBPβ expression were also with high MYC expression (Fig. 5c). All those results emphasized the important roles of MYC and C/EBPβ in heart failure development.

### MYC and C/EBPβ target genes are down regulated in failing heart tissues

**Fig. 6:**
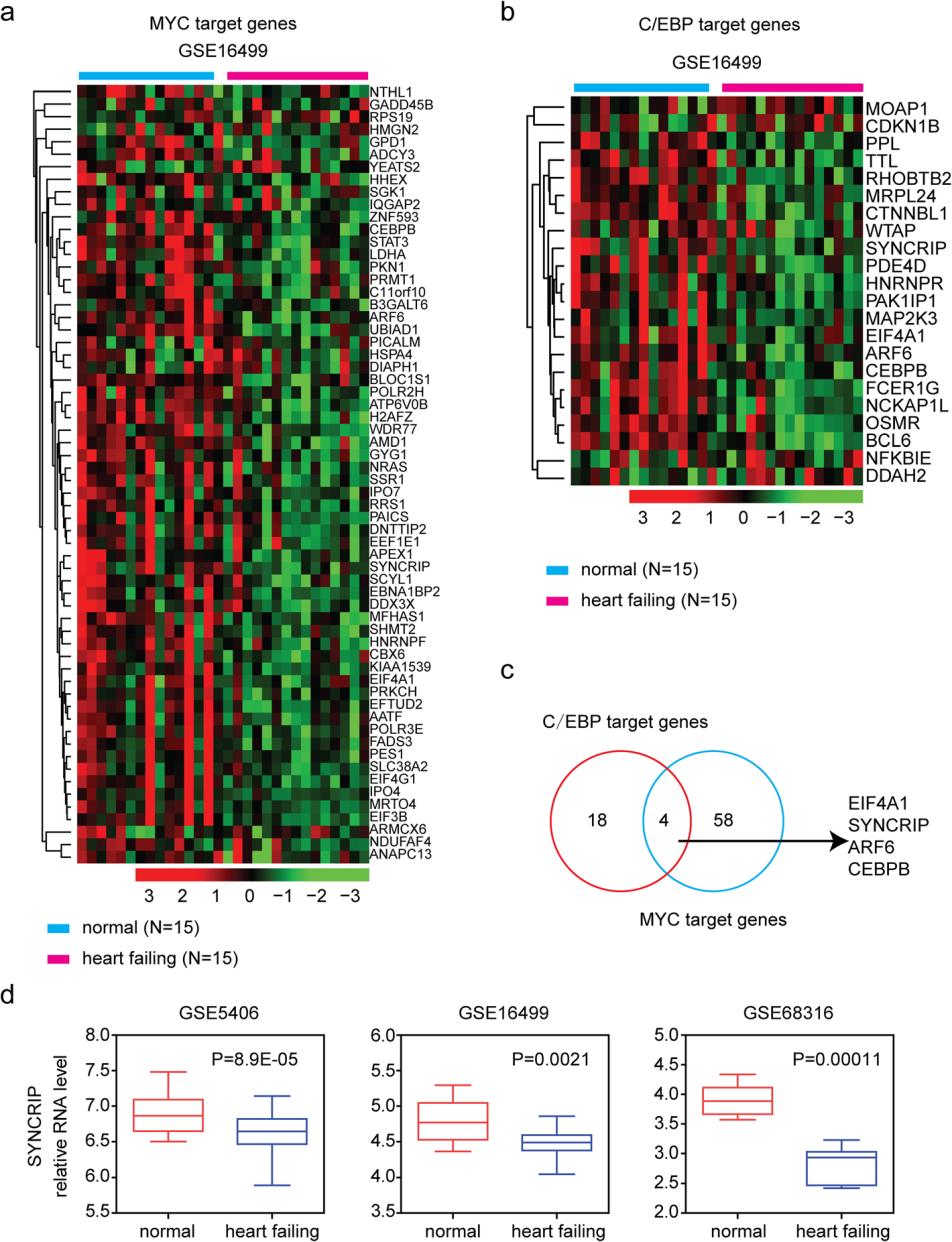
MYC and C/EBPβ target genes are down regulated in failing heart tissues. **a** Heatmap demonstrated the expression profile of MYC target genes in failing heart and normal heart tissues in GSE16499 dataset. Genes up-regulated (red), down-regulated (blue) and moderately regulated (black) were delineated. **b** Heatmap demonstrated the expression profile of C/EBPβ target genes in failing heart and normal heart tissues in GSE16499 dataset. **c** Venn diagram depicted the four overlapped MYC and C/EBPβ target genes. **d** Box plots showed the expression levels of SYNCRIP

Transcription factors are usually the master regulators of disease and regulate multiple target genes. In the GSEA assay, we identified 62 MYC target genes and 22 C/EBPβ target genes. Consistent with the decreased expressions of MYC and C/EBPβ in failing heart tissues, MYC target genes were down regulated in failing heart tissues, compared with normal heart tissues (Fig. 6a). C/EBPβ target genes were also suppressed in failing heart tissues in GSE16499 dataset, as demonstrated in the heatmap (Fig. 6b).

Interestingly, we found that some genes, for example, EIF4A1, SYNCRIP, ARF6 and C/EBPβ, were both MYC and C/EBPβ downstream target genes (Fig. 6c). We showed that SYNCRIP gene expression was particularly down regulated in failing heart tissues in all GSE5406, GSE16499 and GSE68316 datasets (Fig. 6d).

### The MYC and C/EBPβ mediated transcriptional networks

**Fig. 7:**
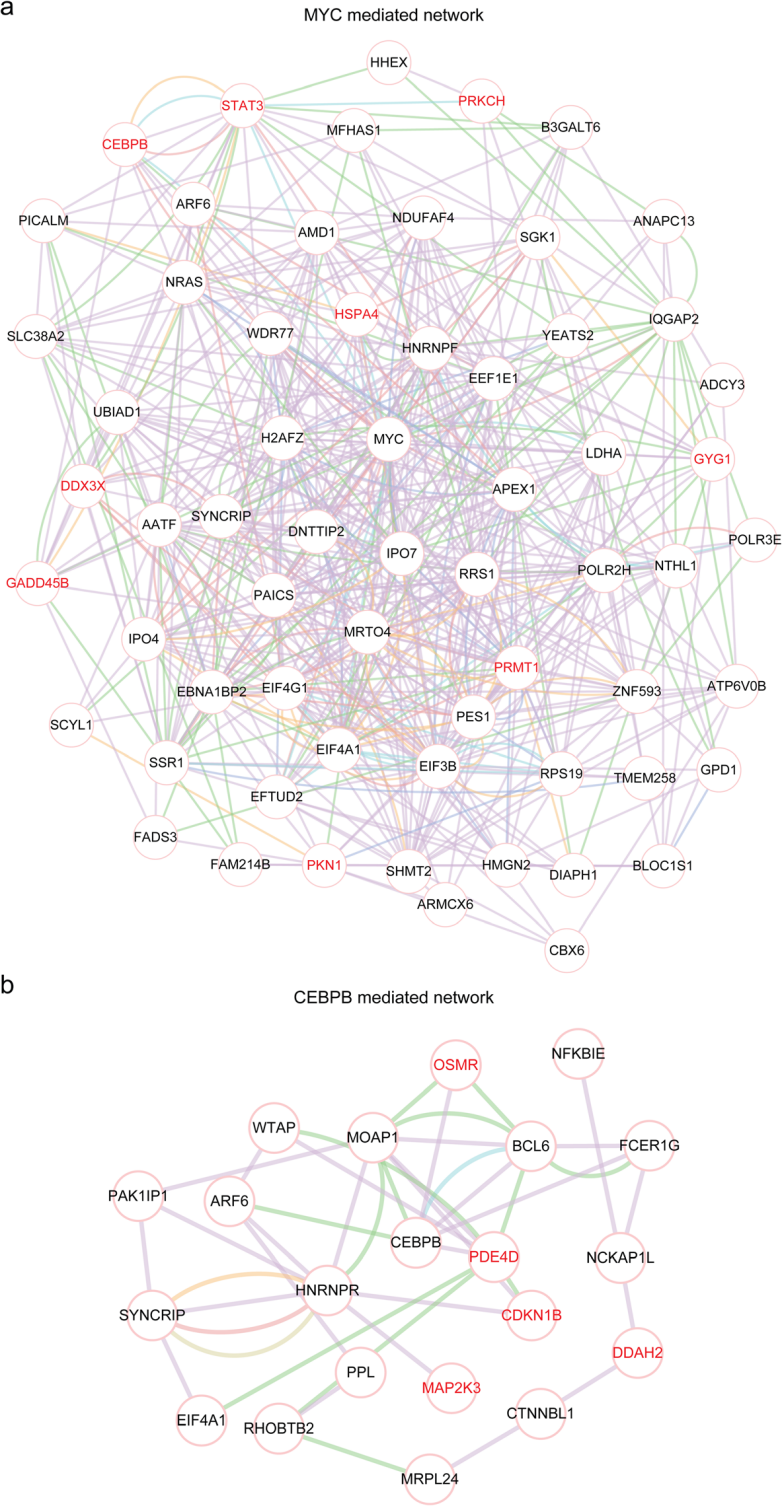
The MYC and C/EBPβ mediated transcriptional networks. **a** MYC mediated regulatory network was created by cytoscape using MYC target genes. The red ones implied that those genes were previously reported involving the development of heart failure. **b** C/EBPβ mediated regulatory network was created by cytoscape using C/EBPβ target genes

To further explore MYC and its connection to downstream target genes, the MYC mediated regulatory network was constructed using Cytoscape. As expected, as a MYC target gene, C/EBPβ was connected with MYC through the transduction of multiple genes (Fig. 7a). Furthermore, through literature research, we found that some MYC target genes were previously reported involving the development of heart failure, including STAT3 [30], PRMT1 [31], PRKCH [32] and HSPA4 [33] (Fig. 7a).

Similarly, the C/EBPβ mediated regulatory network was constructed (Fig. 7b). Some C/EBPβ target genes, for example, OSMR [34], MAP2K3 [35] and CDKN1B [36] also have been studied in heart failure developmental progress (Fig. 7b). All those results highlighted the importance of MYC, C/EBPβ and their downstream target genes in heart failure development. The functions of other MYC and C/EBPβ target genes should be further studied to reveal their connections with heart failure.

## Discussion

Complex diseases like heart failure are often involving malfunctions of multiple genes. Disease related genes detected by different microarray studies are often highly inconsistent, even when there is not much technical noise [7]. As described in the present study, compared with normal heart tissues, there are 2184 differentially expressed genes in failing heart tissues in GSE5406, 1644 genes in GSE16499 and 3477 genes in GSE68316 dataset. However, only 90 genes are commonly up/down regulated in all three datasets. Those differentially expressed genes are associated with MYC and C/EBPβ transcription factors, metabolism signaling pathways and insulin signaling pathway. The converged transcription factors or signaling pathways may have particularly significant roles in heart failure development than single gene.

Indeed, C/EBPβ, MYC and their target genes are all down regulated in failing heart tissues. C/EBPβ is a master regulator in the development of heart failure [9]. Also, some C/EBPβ target genes, for example, OSMR [34], MAP2K3 [35] regulate the heart failure developmental progress. The functions of MYC in the regulating of heart failure development are rather complicated. Previous report suggests that inhibition of MYC is a potential therapeutic approach in the treatment of hypertrophic cardiomyopathy [29]. However, we observe the down regulation of MYC expression in failing heart tissues. MYC target genes are also decreased in failing heart tissues. The inconsistence further emphases the complex transcriptional network regulated by MYC and the complex developmental progress of heart failure.

The aim of the current study is to identify the molecular signaling pathways and transcription factors involving failing heart development. By comparative analysis, our results provide the changed expression profiling of metabolism signaling pathway, insulin signaling pathway, transcription factors MYC and C/EBPβ in the development of heart failure. However, there are certain limitations to the current study. The conclusions were drawn from published databases and lack of further functional validation in failing heart tissues. Therefore, quantitative PCR would have been performed to validate the enriched MYC and C/EBPβ genes in failing heart tissues. Furthermore, the precise mechanisms of MYC and C/EBPβ in heart failure development require further elucidation by MYC and C/EBPβ knockout mouse. Nevertheless, our analysis suggests that transcription factor MYC and C/EBPβ play critical roles in heart failure developmental progress.

## Conclusions

Metabolism signaling pathway, insulin signaling pathway, transcription factors MYC and C/EBPβ were inhibited in heart failure developmental progress.

## Data Availability

The datasets used and analyzed during the current study are available from the corresponding author on reasonable request. Accession numbers of the datasets used in current study are GSE5406, GSE16499 and GSE68316 in Gene Expression Omnibus.

## Abbreviations

GEO: Gene expression omnibus
KEGG: Kyoto encyclopedia of gens and genomes
DAVID: The database for annotation, visualization and integrated discovery
GSEA: Gene set enrichment analysis
FDR: False discovery rate
